# Increased Hair Cortisol Levels Is Associated With Coronary Artery Calcification in Middle-Aged Women but Not in Men

**DOI:** 10.1177/24705470261476161

**Published:** 2026-08-11

**Authors:** Fredrik Iredahl, Elvar Theodorsson, Tomas Faresjö, Michael P. Jones, Andrea Lebena, Per Dahlqvist, Åshild Faresjö

**Affiliations:** 1 Primary Health Care Center Åby, Department of Health, Medicine and Caring Sciences, 571308 Linköping University, Linköping, Sweden; 2 Wallenberg Center for Molecular Medicine (WCMM), 59591 Linköping University, Linköping, Sweden; 3 Department of Biomedical and Clinical Science, Clinical Chemistry, 59591 Linköping University, Linköping, Sweden; 4 Department of Health, Medicine and Caring Sciences, General Practice, 571308 Linköping University, Linköping, Sweden; 5 School of Psychological Sciences, 7788 Macquarie University, Sydney, NSW, Australia; 6 Department of Health, Medicine and Caring Sciences, Society and Health/Public Health, 571308 Linköping University, Linköping, Sweden; 7 Center for Social and Affective Neuroscience, Department of Biomedical and Clinical Sciences, 59591 Linköping University, Linköping, Sweden; 8 Department of Public Health and Clinical Medicine, 59588 Umeå University, Umeå, Sweden

**Keywords:** population-based study, coronary artery calcification, chronic stress, cortisol, sex, cardiovascular risk factors

## Abstract

**Background:**

Chronic stress exposure has been linked to cardiovascular risk factors and cardiovascular diseases, but not to coronary artery calcification (CAC). Hair cortisol concentrations is a potential biomarker of chronic stress. Atherosclerosis is the predominant cardiovascular disease and the leading cause of death in developed countries. Coronary artery calcification is a sign of atherosclerosis in the coronary arteries and predicts coronary heart disease. Most men and women are to some degree affected by coronary artery calcification at the age of 65.

**Objectives:**

To analyse if increased hair cortisol levels are associated with CAC in a middle-aged population with special focus on women. Further, to analyse the relative importance of HCC for CACs in relation to established cardiovascular risk factors.

**Methods:**

Hair samples, cardiac computed tomography data, health questionnaires, and anthropometric measures were collected in 4821 men and women aged 50-65 years, from the Swedish Cardio Pulmonary bioImage Study. A competitive radioimmunoassay was used to analyze cortisol levels in extracts of hair. Correlation, descriptive, regression and path analyses were applied.

**Results:**

A significant association between high HCC and high CAC score was found among women (p=0.007), but not among men. HCC was significantly directly associated with a higher CAC score among women, as were the other classical cardiovascular risk factors.

**Conclusion:**

The amount of calcified plaque (CAC score) in the coronary arteries of women was significantly associated with higher cortisol levels in hair, while this association was not observed in men.

## Introduction

Atherosclerotic cardiovascular disease is the predominant cause of death both in men and women in developed countries.^1-6^ Longstanding stress has been linked to cardiovascular disease (CVD) in epidemiological, behavioural, and mechanistic studies.^3,7-9^ Although cortisol has been associated with coronary atherosclerosis, its role in structural and functional cardiac disease remains unclear.^10^ Most men and women are to some degree affected by coronary artery calcification at the age of 65. Coronary artery calcification (CAC) is a radiological sign of atherosclerosis in the coronary arteries and predicts the risk of coronary heart disease. Higher coronary artery calcification score (CACs) has been observed in people with chronic life stress,^1^ and also in depression,^2^ which trigger similar physiobiological mechanisms as external stressors. Being a widespread and progressive disease, subclinical atherosclerosis affects most people to some degree by the age of 65.^11^ However, men have approximately twice the lifetime risk of myocardial infarction (MI) compared to women, even after adjusting for conventional risk factors, and women are generally affected 5–7 years later than men.^12^ This is consistent with the observation that men have more coronary calcification than women of the same age.^13,14^ The prevalence in women 65 years old correspondig to men 11-13 years younger, these associations remained after multivariable adjustment.^14^

Risk factors for atherosclerosis are similar for men and women, but some risk factors, like diabetes, hypertension, and smoking, seem to have a somewhat more substantial impact in women.^3,15^ Coronary atherosclerosis can be detected through imaging, i.e, the coronary artery calcification score (CACs) indicates coronary atherosclerosis and predicts coronary heart disease (CHD).^16^ The issue of cortisol sensitivity is another relevant aspect i.e that the individual glucocorticoid sensitivity may also play a role in the effect of long-term cortisol exposure.^17^ External stressors activate the sympathetic nervous system and the hypothalamic-pituitary-adrenal (HPA) axis, increasing catecholamine and cortisol levels.^18,19^ The cortisol response occurs when mental or environmental stress initiates the release of cortisol by activating corticotropin-releasing factors and arginine vasopressin neurons in the paraventricular nucleus of the hypothalamus. This leads to the release of adrenocorticotropic hormone from the pituitary gland, which triggers the release or response of cortisol from the adrenal glands. A definition of the concept “Chronic stress” could be: chronic stress is a persistent, overwhelming sense of strain that lasts over weeks or months and includes a long-term activation of the stress response system. Supraphysiological exposure to cortisol and other stress hormones can disrupt numerous bodily processes. This puts the exposed individual at a higher risk of many health problems.

Several potential mechanisms have been discussed regarding cortisol’s role in developing CVD, including its contributions to hypertension, insulin resistance, hemodynamic stress on arterial walls, inflammation, positively associated to BMI, atherosclerosis, waist circumference and thrombosis.^18-22^ Environmental stressors, along with behavioural responses such as low physical activity, a high-fat diet, and smoking, may also contribute to CVD.^8^ Stress activates hormonal responses, including the HPA axis, leading to the release of cortisol from the adrenal cortex. Previous studies have shown associations between increased stress measured by urinary catecholamines and cortisol and plaque burden in coronary vessels, hypertension, and adverse cardiovascular events.^23,24^ Long-term exposure to elevated cortisol levels can increase the risk of cardiovascular disease and death.^25^ Cortisol levels can be used as a biomarker of stress when measured in saliva, blood, or urine; however, due to the short half-life of cortisol, which circulates for approximately 1.5 hours and large variations due to circadian rhythm and brief stressors, these methods offer limited information about long-term cortisol secretion.^26,27^ Over the past decade, the use of hair cortisol concentrations (HCC) as a potential biomarker for long-term stress has gained prominence, providing insight into cumulative cortisol levels over several months. HCC is stable and reliable, and can offer valuable insights into the role of hyperactivity of the HPA axis.^28-34^ The concentrations of cortisol deposited in the hair are retrospective reflections of the biologically active plasma concentrations of the hormone during the period of growth.^35^ Scalp hair grows approximately 1 cm per month, enabling a timed retrospective evaluation of long-term cortisol levels using only one sample.^36^ Hormone analysis in hair has the additional advantage of circumventing difficulties in determining the biologically active fraction, while avoiding large variations cortisol due to circadian rhythm when collecting samples from an individual. Hair samples are stable at room temperature for months and years, facilitating long-term storage.^27,35^

Sex differences in CVD risk have become more clearly recognised, with stress emerging as a potential predictor of CVD risk in women. In this context, women and men may have different responses to stress. This difference might be seen concerning CAC as well, one study found that urinary cortisol was associated with a greater change in CAC in women but not in men.^37^ For example, studies have shown that women with CVD are more susceptible to mental stress-induced myocardial ischemia, which increases their risk of mortality and other adverse cardiovascular events.^38-41^

Mental or environmental stress initiates the release of cortisol by activating corticotropin-releasing factors and arginine vasopressin neurons in the paraventricular nucleus of the hypothalamus. This leads to the release of adrenocorticotropic hormone from the pituitary gland, which triggers the release of cortisol from the adrenal glands.^42^

Research also demonstrates an association between higher cortisol reactivity to stress and the progression of CAC.^26^ This data could support the idea that cortisol reactivity, an indicator of HPA function, may be one of the mechanisms through which psychosocial stress affects CVD risk.^41^ A study of healthy older adults found that long-term, but not current, psychological distress is associated with more severe CAC. Participants experiencing both long-term distress and increased cortisol response were particularly at risk for severe calcification in this study focused on older people.^42^

**Aim:** To investigate whether long-term cortisol levels as measured by HCC were associated with increased coronary artery calcification (CACs) in a middle-aged general population, with special focus on women. Analyse the relative importance of HCC for CACs in comparison to established cardiovascular risk factors. We hypothesise that increased levels of the stress hormone cortisol, as measured by HCC, is associated with higher CAC score in a middle-aged population and that this possibly differs between men and women.

## Material and Methods

### Study Design and Participants

The study is based on the SCAPIS study, a prospective national observational project involving 30154 individuals aged 50-64 years, randomly selected from the Swedish population. The primary goal of SCAPIS is to explore cardiovascular disease mechanisms and identify new biomarkers. Participants were recruited from six Swedish university hospitals: Gothenburg, Linköping, Malmö/Lund, Stockholm, Uppsala, and Umeå. Data collection occurred across six sites in Sweden from November 2013 to November 2018, following a detailed clinical examination protocol that lasted over 2–3 days. This included questionnaires on medical history, lifestyle, and hair sample collection at two sites.The same study protocol was used for all study sites; however, it was also possible to add optional examinations depending on local research interests. Further details are provided in the Supplementary Data section.^43^

An additional data collection took place within the national SCAPIS study at the Linköping and Umeå sites, focusing on measuring hair cortisol levels as a biomarker for long-term stress. This sub-study involved a total of 4921 hair samples that were analysed the 3 cm closest to the scalp representing 3 months backwards in time, since hair grows, approximately 1 cm/month. The great majority of the hair samples in this study were at least 3 cm long, i.e. representing the last 3 months. However, there are some differences between human races in hair growth. Asian hair grows a little faster and African hair grows a bit slower than Caucasian hair, in this study almost all had Caucasian hair.^36^

In our previous research analyzing HCC we have constantly found that males tend to have higher HCC levels than women, that is why we choose to analyse separately for man and women.^31,44^

The total hair samples collection comprised N=3462 from Linköping and N=1459 from Umeå, included n=1712 males and n=3209 females. The overall dropout rate was 34.9% (n=2643 of N=7564 participants), drop-out was mainly because hair was too short; only a few participants refused to provide samples. For the hair sampling an earlier analysis of non-attendees, i.e. men without hair samples compared to those with samples, in the SCAPIS cohort showed no significant differences in educational level or cardiovascular risks among those with hair samples and those without (mainly due to baldness or to short hair).^41,45^

For the proportion of individuals with a prior history of CVD, cancer or COPD among individuals invited to the SCAPIS pilot study was similar among participants and non-participants.^45^ However, for sociodemographic factors a particularly low participation rate was found among individuals born outside Europe, living single in a low SES area, having low education, being outside the labor market and having low income, but this data derive from the SCAPIS pilot study.^45^ All samples were analysed at the research laboratory of clinical chemistry at the University Hospital in Linköping, Sweden.

An extensive questionnaire covered background data on lifestyle, social, and psychosocial factors, as well as self-reported physician-diagnosed conditions. Socioeconomic status was based on education and divided into low, medium, and high; smoking was divided into yes now and earlier regular smoker vs. no smoker. Age was divided into three age groups based on age at study inclusion: i) 50-54, ii) 55-59, and iii) 60-65 years. Marital status was divided into i) single/unmarried and ii) married/cohabited. Questions regarding women’s use of hormone replacement therapy during menopause were classified as i) yes or ii) no. Physicians’ diagnoses of hypertension and elevated plasma cholesterol concentrations, high-density lipoprotein (HDL), low-density lipoprotein (LDL), and triglyceride concentrations were reported and into i) yes or ii) no. Diagnosis of diabetes and impaired glucose tolerance was divided into a 3-grade scale: i) normoglycemia, ii) diabetes, and iii) impaired glucose tolerance. The question measuring perceived stress was formulated as; “By stress, we mean feeling tense, irritable, or anxious, or having sleep difficulties, as a result of conditions at work or at home. Did you experience this?”, with responses (0–4) ranging from 0 = “Never experienced this” to 4 = Constant stress the latest five years” and constant life stress during the latest 1–5 years (yes/no). This question about self-reported stress was divided into a 3-grade scale of i) always stressed, ii) stressed now and then, iii) never stressed. Self-reports of physical activity during the previous 12 months were dichotomised as i) none or little activity or ii) regular physical activity. Further details are provided in the Supplementary Data section.

**Measuring coronary artery calcification (CAC) in SCAPIS** was done by professional personnel at each site according to standard procedure, described elsewhere.^5,43^ Briefly, the amount of calcified plaque in the coronary arteries (CAC) was measured by using a multi-slice computed tomography scanner (Siemens Acuson S2000 ultrasound scanner equipped with a 9L4 linear transducer, both from Siemens, Forchheim, Germany.^3,41^ The two-dimensional greyscale ultrasound image was analysed to determine plaque size and the number of plaques. The calcium content in each coronary artery was measured and summed to produce a total coronary artery calcification score (CACs) in accordance with international standards.^16^ The area of calcification of each 3-mm slice was multiplied by an intensity factor and summed across slices for the whole coronary artery tree to a CAC score according to Agatston.^46^Due to the rather wide variation in CAC score, total score, the distribution was skewed, and a large number of participants with CACs=0 in this study, three groups were defined: CAC score=0 (no calcification), CAC score=1-99, and CAC score>100; these latter two represent the presence of detectable calcification and being compared with CACS = 0.^47,48^ Missing data for the measurements of CAC score were n=70 for women and n=67 for men in our sub study.

### Analyzing Cortisol Levels in Hair

Hair, with a minimum length of 3 cm, was cut from the vertex posterior of the scalp as close to the skin as possible by trained staff, as recommended by the Society of Hair Testing (SoHT).^43^ Hair in this area has a normal growth rate.^49-53^ The hair samples were dissolved in a radioimmunoassay buffer and analyzed as described by Mörelius et al.^54,55^ A competitive radioimmunoassay (RIA) was used to analyze cortisol concentrations in the hair extract, as described by Yang et al.^56,57^ Previous work has shown that hair samples cut down to 5 mg or more are needed toachieve an inter-assay coefficient of variation below 8% for hair extraction and cortisol measurement.^54,55^ The intra-assay coefficient of variation for the radioimmunoassay was 7% at 10 nmol/L.

### Risk Factors and Anthropometric Measures

Disease and risk factor data were included as self-reports in the SCAPIS questionnaire.^6^ These included reports of physicians’ previous diagnoses of type 2 diabetes, high cholesterol levels, and hypertension. All chosen covariates have been selected as previously scientific well-documented cardiovascular risk factors.^6,14,31^ The laboratory blood measurements in this study were performed at two separate but synchronised standardised routine laboratories. Triglycerides, HDL and LDL were classified as being within or above/below the reference range.

Body mass index (BMI) results were categorized into normal BMI (18.5-25), overweight (BMI >25-30), and obese (BMI >30). Waist circumference was divided into five grades: 60-79 cm, 80-87 cm, 88-94 cm, 95-101 cm, and 102-168 cm according to standard procedure. An open question about the use of glucocorticoid medication was also asked. In the total cohort, n=478 participants reported using some form of corticosteroids, such as ointments/creams, inhalers, tablets, or a combination of these. Participants reporting regular use of glucocorticoid ointments or/cream (n=100) had significantly higher UTHCC (p=0.001). These were omitted from further analysis since hair contamination by exogenous hydrocortisone cream is a potential confounding factor. Median values for female (n=72) before omitting are 25.42 pg/mg, and after omitting are 25.13 pg/mg UTHCC. For male (n=28), median values before omitting are 32.98 pg/mg, and after omitting are 32.57 pg/mg UTHCC.

### Statistics

**Figure 1.**
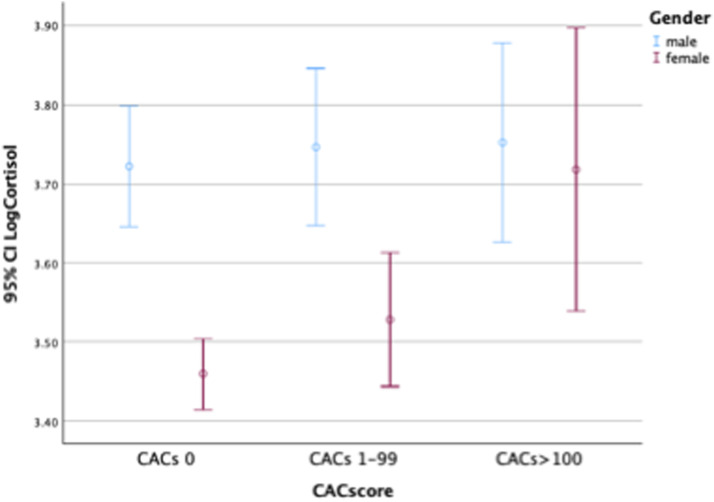
A three-grade CAC-score and means and 95% confidence intervals CI) of HCC based on logarithmic cortisol (LogHCC) values for men and women. Mean LogHCC increased with a higher CAC score for women only. Thus, women with CACS >100 showed significantly higher HCC than women with CACS=0 (p=0.007), and CACS= 1-99 (p=0.08)

Untransformed hair cortisol concentrations (UTHCC) are presented as median values with the interquartile range (the difference between the third and first quartiles, similar to a standard deviation). Categorical data are shown as counts and percentages. Normality tests using the Shapiro-Wilk method indicated a non-normal distribution of HCC, characterized by a long right tail, prompting a logarithmic transformation (LogHCC) for statistical analysis. LogHCC was compared across CACs, lifestyle factors, and diagnoses using analysis of variance, with the overall test of mean equality reported (see Figure 1). LogHCC served as the dependent variable. The CAC total score, were divided into three categories: CAC=0, CAC=1-99, and CAC>100. These were analyzed in relation to demographic, lifestyle, biological, and anthropometric factors. In regression analyses, lifestyle factors, waist circumference, and high triglycerides were preferred over BMI and high LDL(however high LDL is preferred over triglycerides in the final regression model for men due to p-value 0.07 compared to 0.61 in model 3), respectively, due to the lower correlation with LogHCC in both sexes. In all regression models odd ratio (OR) was use to show effect size.

**Figure 2.**
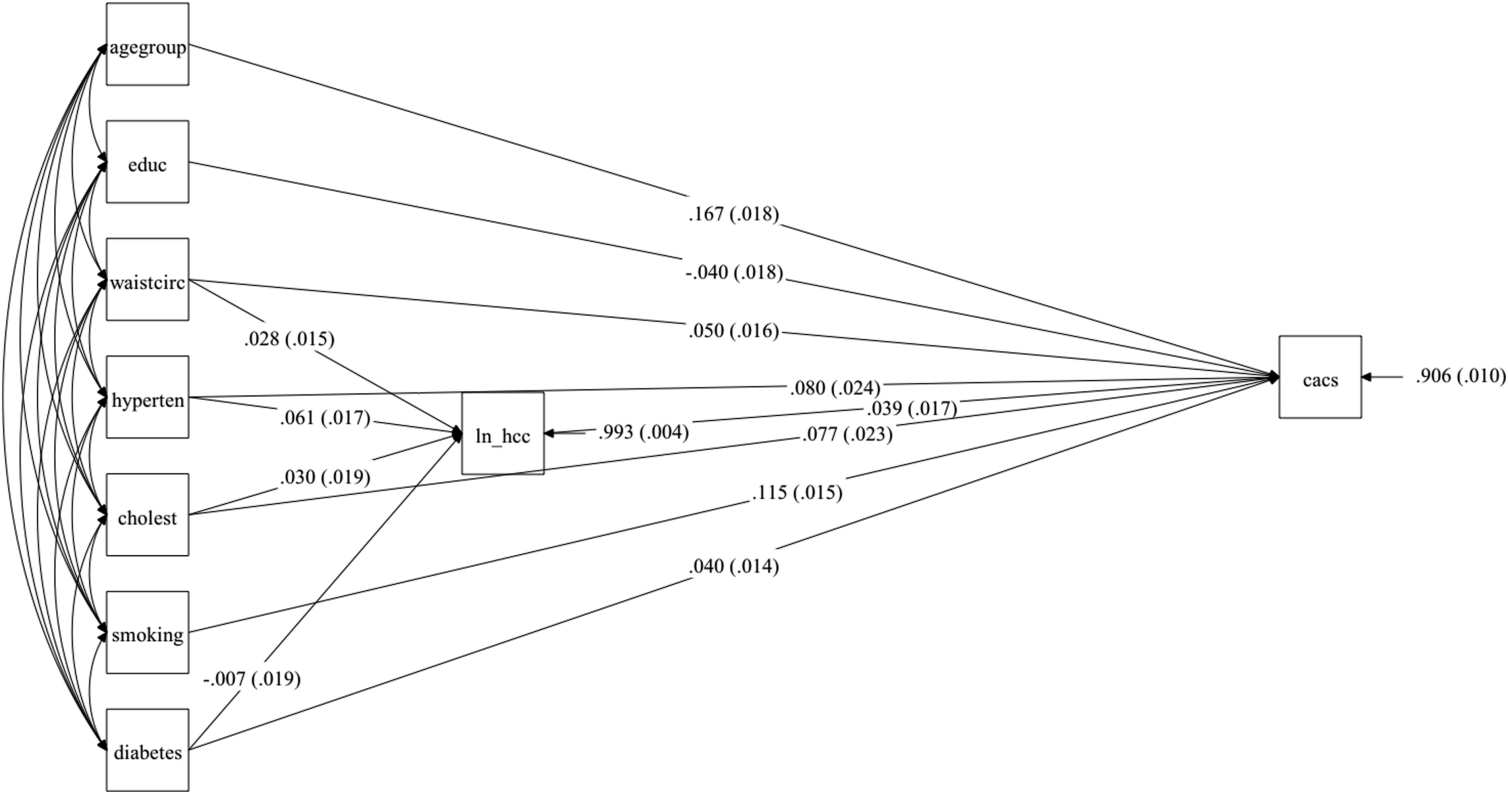
Pathway analysis of direct and indirect associations of different cardiovascular risk factors on CAC for women, also, including LogHCC as a potential risk factor. Association is expressed as r-values and (p-values).Log HCC was significantly directly associated with a higher CAC score among women, as were the other classical cardiovascular risk factors. Some risk factors, like waist circumference, hypertension, and high cholesterol, also expressed a weaker association with high CAC score through LogHCC (Ln_HCC), but a stronger direct association with CAC>100. Diabetes seems to have a negative association with HCC and a stronger positive directly with high CAC Score

To correct for covariates multiple ordinal regression analysis was performed with the 3-grade CAC score as the dependent variable. Regression results are reported as odds ratio (OR) [95% confidence interval] and two-tailed p-value. The ordinal regression model was used to analyse risk factors associated with increasing CAC score categories, with LogHCC as the primary predictor. To identify covariates,social and biological and health related factors were compared across CAC categories in men and women separately. Other factors included demographic characteristics (model 1), lifestyle factors such as exercise, perceived stress, smoking, and waist circumference (model 2), and finally, health-related issues such as diabetes, hypertension, and high cholesterol (model 3). The ordinal regression included variables that were statistically significant and correlated to CACs in Spearman’s correlation analysis (see Table 3), as well as other variables that were demonstrated to be associated with UTHCC like median UTHCC above the general Median (see Table 2). The final regression model comprised the statistically significant factors from the previous 3 models (see table S2 and S3). Separate analyses were undertaken for women and men. The main purpose of the 3 steps was to evaluate covariates systematically. A Path analysis was undertaken to quantify what percentage of the associations between the factors and CAC score operated indirectly via hair cortisol concentration and what percentage represents a direct association. This analysis was primarily made for females and shown in Figure 2. Additionally, a sensitivity analysis was undertaken in participating women in the highest quintiles (Q) of UTHCC to investigate what factors differ between those in the highest Q compared to those in the lower Q (Table S1). All descriptive, correlational and regression modelling was undertaken using SPSS version 29. The path modelling was undertaken using MPlus version 8. A p-value p<0.05 was considered significant in all analyses. No allowance for multiple hypothesis testing has been undertaken and our results are therefore open for replications.

## Results

The study included N=4821 participants, of whom 65% (n=3137) were women. The distribution and characteristics of the study population are shown in table 1.

**Table 1.** Distribution of the Study Population

Variables	Women (n=3137)		Men (n=1684)	
	%	n	%	n
**Age-groups**				
50-55 years	35.1	1100	34.3	577
56-59 years	32.2	1011	32.5	548
60-65 years	32.7	1026	33.2	559
**Education**				
Low	7.5	234	9.0	146
Medium	44.6	1398	49.7	810
High	45.7	1433	41.3	673
**Civil status**				
Unmarried/single	21.8	669	20.4	331
Married/cohabited	76.3	2393	79.6	1292
**Smoking**				
Yes now and preivious	42.6	1335	38.2	615
No	53.7	1685	61.8	996

Median HCC increased with a higher CAC score for the women only, as illustrated in Figure 1. Thus, women with CACS >100 had significantly higher HCC than women with CACS=0 (p=0.007).

A significantly higher UTHCC was observed in individuals with obesity, specifically among women with a waist circumference over >88 cm (p<0.001), compared to those below 88 cm. Among men, those with a waist circumference above >102 cm showed increased UTHCC compared to those below, but this difference was not statistically significant (Table 2). Participants with hypertension and high cholesterol levels in both women and men exhibited significantly higher UTHCC values (p<0.001 and p=0.006 in women, respectively). Men with hypertension also had significantly higher UTHCC than those without (p=0.001). Similarly, increased cholesterol levels were associated with higher UTHCC (p=0.005). Individuals with type 2 diabetes showed significant differences in UTHCC compared to those with normal glucose levels, p=0.003 in women and p=0.02 in men (Table 2).

**Table 2.** Biological and Anthropometric Distribution Among the Study Population for Women and Men in Relation to Untransformed Hair Cortisol Concentration (UTHCC)

Variables	Women (n=3137)		UTHCC median (25.13 pg/mg)1 pg/mg	p-value	Men (n= 1684)		UTHCC median (32.92pg/mg)1 pg/mg	p-value
CAC ‘s	%	n	Median (IQR)2		%	n	Median (IQR)2	
CAC=0	71.9	2211	24.45 (39.37)	​	48.5	784	32.06 (52.59)	​
CAC =1-99	22.5	691	25.89 (47.20)	0.08	33.6	545	31.61 (52.32)	​
CAC >100	5.7	174	**29.73** (78.18)	0.007	17.9	288	**34.65** (60.94)	0.72
**BMI**								
Underweight	0.8	24	16.66 (14.97)	​	0.2	3	33.34 (31.65)	​
Normal weight	42.7	1329	24.16 (38.30)	​	29.1	486	32.39 (60.16)	​
Overweight	36.6	1138	25.06 (41.44)	​	49.3	823	30.94 (48.99)	​
Obese	19.9	618	**28.65** (55.94)	<,001	21.3	356	**38.04** (60.50)	0.38
**Waist Circumference**								
**>88 cm W3 (>102) M**	46.9	1472	**27.32** (47.26)	0.002	36.6	616	**34.11** (56.99)	0.59
**80-88 cm W (94-102)M**	26.5	830	23.53 (41.12)	​	48.8	821	32.00 (50.73)	​
**<80 cm W (<94) M**	26.6	23.31	23.31 (36.89)	​	14.6	246	32.51 (64.64)	​
**HDL** below the reference range value	6.4	200	22.95 (41.69)	0.52	6.5	109	32.45 (53.09	0.87
**HDL** within the reference range (1.0-2.7 mmol/l)	93.6	2933	25.19(42.21)	​	93.5	1574	32.57 (53.73)	​
**LDL** above the reference range	9.0	280	**26.36** (48.18)	0.17	10.7	177	**33.20** (51.12)	0.38
**LDL** within reference range (2.0-5.3mmol/l)	91.0	2835	24.95 (41.35)	​	89.3	1484	32.33 (53.80)	​
**Triglycerides** above the reference range	3.9	123	**28.97** (67.09)	0.36	7.3	123	**40.55** (61.26)	0.55
**Triglycerides** the within reference range (0.45-2.6 mmol/l)	96.1	3010	24.98 (41.31)	​	92.7	1560	32.23 (52.62)	​
**Hypertension**								
Yes	21.2	636	**28.28** (58.52)	<,001	23.3	377	**38.96** (60.53)	0.002
No	78.8	2416	24.33 (39.42)	​	76.7	1240	31.83 (50.64)	​
**High cholesterol**								
Yes	8.3	254	**33.13** (59.20)	0.003	14.0	227	**41.26** (68.55)	0.007
No	91.5	2798	24.83 (40.99)	​	86.0	1390	32.12 (51.86)	​
**Diabetes and impaired glucose**								
Diabetes	5.1	158	**32.66** (58.81)	0.01	9.3	157	**40.90** (65.72)	0.03
Impaired glucose	13.6	426	25.34 (39.50)	0.68	15.3	257	28.01 (46.07)	0.38
Normoglycemia	81.3	2590	24.62 (41.44)	​	75.4	1267	32.58 (52.84)	​
**Use of oestrogen menopause yes**	20.1	607	25.32 (44.59)	​	-	-	-	​
No	79.9	2416	24.98 (41.39)	0.73	-	-	-	​

1. The bold mark in table is the HCC median above the general median in women and men in SCAPIS.

2. IQR measure dispersion, between the 25th percentile and 75th among HCC value in each variable.

3 W= women, M= men.

A CAC score exceeding 100 was significantly more prevalent among men (17%) than among women (5.5%) (p<0.001). The percentage of participants with CACs >100 increased with age in both sexes (Table 3). Women participants with CACs >100 were notably more likely to have lower educational level, smoke more, being less physically active, and have a waist circumference >88 cm. These along with having higher low-density lipoprotein levels, hypertension, high cholesterol, and type 2 diabetes, compared to those with lower CAC scores (0-99). Men participants with CACs >100 similarly showed higher rates of smoking, waist circumference >102 cm, elevated low-density lipoprotein and triglycerides, and a greater prevalence of diagnosed hypertension, high cholesterol, and type 2 diabetes compared to those with lower CAC scores (Table 3).

**Table 3.** Distribution of CAC Score Among Women and Men in the SCAPIS Study Population in Relation to Biological and Social Factors

Variables	CACS=0 n=2211	CaCs-1-99 n=691	CACs >100 n=174	p-value 1	Rho/^2^ p-value^3^	CACs=0 n=784	CACs 1-99 n=545	CACs >100 n=288	p-value^1^	Rho2/p-value^3^
	Women	Women	Women			Men	Men	Men		
	%/n	%/n	%/n			%/n	%/n	%/n		
**Age-groups**	​	​	​	<0.001	-.206 <.001	​	​	​	<,0001	-,233 <,001
50-55 years	40.7/900	24.5/169	10.3/18	​	​	44.1/346	31.2/270	19.4/56	​	​
56-59 years	31.8/703	33.2/230	35.1/61	​	​	32.1/252	34.1/186	28.8/83	​	​
60-65 years	27.5/608	42.3/292	54.6/95	​	​	23.7/186	34.7/189	51.7/149	​	​
**Education**	​	​	​	<0.001	-.103 <.001	​	​	​	0.56	-,043 0.09
Low	6.5/141	9.4/64	15.2/26	​	​	7.9/60	9.1/48	10.0/28	​	​
Medium	44.1/952	49.9/338	50.3/86	​	​	48.5/368	50.3/266	51.4/144	​	​
High	49.4/1067	40.7/276	34.5/59	​	​	43.5/330	40.6/215	38.6/108	​	​
**Civil status**	​	​	​	0.05	.040 0.03	​	​	​	0.64	-,017 0.49
Unmarrid/single	20.8/448	23.2/157	27.9/48	​	​	19.3/146	21.5/113	20.4/157	​	​
Married/cohabited	79.2/1710	76.8/520	72.1/124	​	​	80.7/609	78.5/413	79.0/223	​	​
**Smoking**	​	​	​	<.001	-.154 <.001	​	​	​	<0,001	-,131 <,001
Yes now and previously	39.4/842	54.0/357	63.9/108	​	​	32.5/243	38.2/200	51.4/143	​	​
No	60.6/1294	46.0/304	36.1/61	​	​	67.5/505	61.8/323	48.6/135	​	​
**Perceived stress**	​	​	​	0.13	.038/0.004	​	​	​	0.66	.020 0.42
Always Stressed	23.9/511	20.7/139	18.6/31	​	​	16.5/125	16.6/87	15.5/43	​	​
Stressed now and then	72.7/1552	75.2/504	79.6/133	​	​	49.2/588	32.9/394	17.9/214	​	​
Never stressed	3.3/71	4.0/27	1.8/3	​	​	5.9/45	8.0/42	7.2/20	​	​
**Physical activity**	​	​	​	0.001	-.070/<,001	​	​	​	0.11	-,052 0.04
No Exercise or little	57.4/1217	62.6/412	72.0/121	​	​	56.2/419	61.3/313	61.9/167	​	​
Exercise (regular)	42.6/905	37.4/246	28.0/47	​	​	43.8/327	38.7/103	38.1/103	​	​
**Waist circumf.W**	​	​	​	<.001	-,112 <.001	​	​	​	​	​
>88 cm	43.8/967	53.1/367	62.6/109	​	​	-	-	-	-	-
80-88 cm	27.3/603	26.3/182	18.4/32	​	​	-	-	-	-	-
< 80 cm	29.0/640	20.5/142	19.0/33	​	​	-	-	-	-	-
**Waist circumf.M**	​	​	​	​	​	​	​	​	<,001	-,144 <,001
>102 cm	-	-	-	-	-	31.5/247	36.1/197	51.4/148	​	​
94-102 cm	-	-	-	-	-	51.0/400	50.8/277	39.9/115	​	​
< 94 cm	-	-	-	-	-	17.7/137	13.0/71	8.7/25	​	​
**HDL** outside reference range (low)	6.7/39	5.2/39	5.2/9	0.48	,022/0.23	6.4/50	6.4/35	5.9/17	0.95	,005 0.84
**LDL** outside reference range (high)	8.8/194	7.4/51	15.4/26	0.005	-,011/0.55	8.6/67	8.2/44	13.9/39	0.002	-,046 0.06
**Trigly**. Outside reference range (high)	3.6/80	4.3/30	7.0/12	0.08	-,032/0.07	6.5/51	6.2/34	12.2/35	,003	-,058 0.02
**Diagnosed hyperten.**	17.3/373	26.6/180	41.8/71	<,001	-,146 <,001	12.7/96	26.5/139	48.8/113	<,001	-,046 <,001
**Diagnosed high cholestrol**	5.9/128	12.3/183	20.6/35	<,001	-,139 <,001	7.4/56	13.3/70	26.8/74	<,001	-,046 0.06
**Diagnosed Diabetes type 2**	3.7/82	8.0/55	10.0/17	<,001	-,102 <,001	5.4/42	8.6/47	19.1/55	<,001	-,190 <,001
**Diagnosed impaired glycose**	12.5/274	16.0/110	17.0/29	​	​	11.5/90	17.5/95	19.4/55	​	​

1= p-value Ch2 and 2= Spearman’s correlation coefficient (rho), 3= p-value for Spearman’s correlation.

W= women, M = men.

In both sexes, univariate analysis revealed that the median UTHCC was higher among participants with CACs>100 (30.4 pg/mg IQR: 19-94) compared to those with CACs=1-99 (26.2 pg/mg, IQR: 16-63 (p=0.08) and CACs=0 (24.6 pg/mg, IQR: 15-54) (p=0.007). However, this was significant only among women with CACs=0 compared with CACs>100. Although in men, the median UTHCC was not significantly associated with CACs, CACs> 100 (UTHCC 34.52 pg/mg, IQR 19-79) was slightly increased from the general median UTHCC (32.57 pg/mg). Self-reported stress was associated with CACs (p=0.04) among women, but not significantly associated with UTHCC (p=0.8). (Table S1).

Separate multiple ordinal regression analyses for men and women showed that among women, higher LogHCC was significantly associated with increased odds of a higher CAC score, OR 1.10 [1.02-1.19] (p=0.02). Similar patterns were observed concerning CAC score and factors such as age (55-65 years, p<0.001), smoking (p<0.001), waist circumference (p=0.02), type 2 diabetes (p=0.01), diagnosed hypertension (p<0.001), and high cholesterol (p<0.001), (see table 4). Among men, these associations were also present, except there were no statistically significant links between higher LogHCC or waist circumference and a higher CAC score (Table 5).

**Table 4.** The Final Regression Model Comprised the Statistically Significant Predictors From the Previous 3 Models. See Table S2. Separate Analyses Were Undertaken for Women and Men. Possible Risk Factors for Higher CAC Score Among SCAPIS Female Study Population. Dependent Variable CACs score=0, CACs score=1 (1-99), and CACs Score >100

Factors	Wald	p-value	OR	95% confidence interval lower bound	Upper bound
Log-cortisol	5.64	0.03*	1.08	1.01	1.17
Education low vs high	2.68	0.10	1.31	0.59	1.80
Education medium vs high	2.06	0.15	1.14	0.61	1.37
Age 60-69 years vs <55 years	77.53	<0.001*	2.64	2.13	3.28
Age 55-59 years vs <55 years	23.28	<0.001*	1.73	1.38	2.16
Exercise: No vs Yes	1.17	0.28	1.10	0.92	1.32
Smoking: Yes vs No	35.71	<0.001*	1.70	1.43	2.02
Waist circumference	​	​	​	​	​
>88 cm vs <80cm	5.24	0.02*	1.30	1.04	1.62
80-88 cm vs <80cm	0.50	0.48	1.09	0.86	1.39
Diagnosed Diabetes*	5.58	0.02*	1.54	1.08	2.20
Diagnosed impaired glucose*	1.27	0.26	1.15	0.90	1.46
Diagnosed Hypertension*	13.49	<0.001*	1.46	1.19	1.79
Diagnosed High cholesterol*	9.47	<0.001*	1.57	1.18	2.10

Final model intercept: -2 log likelihood: 3938.73, df:13, p-<0.001. Cox and Snell: 0.085 and Nagelkerke: 0.111.

*vs not diagnosed with indicated condition.

**Table 5.** The Final Regression Model Comprised the Statistically Significant Predictors From the Previous 3 Models See Table S3. Separate Analyses Were Undertaken for Women and men. Possible Risk Factors for Higher CAC Score Among SCAPIS *Male* Study Population: Dependent Variable CACs score= 0, CACs score= (1-99), and CACs Score >100

Factors	Wald	p-value	OR	95% confidence interval lower bound	Upper bound
Log-cortisol	0.080	0.77	0.99	0.90	1.08
Age 60-69 years vs < 55	45.06	<.001*	2.36	1.84	3.04
Age 55-59 years vs < 55 years	12.90	<.001*	1.57	1.23	2.00
Smoking: Yes vs No	5.56	0.02*	1.28	1.04	1.58
Waist circumference	​	​	​	​	​
>102 vs > 102	1.92	0.17	1.26	0.91	1.73
94-102 cm vs > 94	0.25	0.61	1.08	0.80	1.46
LDL high vs within reference value	2.38	0.12	0.76	0.53	1.08
Diagnosed Diabetes*	14.55	0.001*	2.07	1.42	3.01
Diagnosed impaired* glucose	4.34	0.04*	1.35	1.02	1.80
Diagnosed Hypertension*	24.82	<.001*	1.92	1.49	2.67
Diagnosed High cholesterol*	17.20	<.001*	1.95	1.42	3.01

Final model intercept: -2 log likelihood: 2886.37. df:12, p-<,001. Cox and Snell: 0.126 and Nagelkerke: 0.145.

*vs not diagnosed with indicated condition.

A path analysis in women indicated that LogHCC was significantly directly associated with a higher CAC score (b=0.039, SE=0.017, p=0.03), along with other traditional cardiovascular risk factors (see Figure 2). None of the indirect pathways connecting risk factors and CAC score were statistically significant (all p>0.05) and showed small effects, with the largest, for hypertension, accounting for just 2.4% of the total relationship between that risk factor and CAC score category. This implies that the cardiovascular risk factors identified likely influence CVD independently of, rather than through, increased hair cortisol levels.

## Discussion

The main findings in this study indicates that higher hair cortisol concentrations, a marker of long-term stress, are significantly associated with increased coronary artery calcification (CAC) score in women but not in men. This sex-specific association between long-term stress and CAC score is unexpected and warrants further research.

One possible explanation is that women develop cardiovascular diseases about 10 years later than men, a pattern supported by several studies.^11,14^ Therefore, men may have already been exposed to traditional cardiovascular risk factors by midlife, reducing the relative impact of long-term stress as an additional risk. Although risk factors for atherosclerosis are similar for men and women, but some risk factors, like diabetes, hypertension, and smoking, seem to have a somewhat more substantial impact in women. In a previous report from the SCAPIS study, sex differences in prevalence and characteristics of atherosclerosis were displayed. The main results showed that men had higher prevalence of imaging-detected carotid and coronary atherosclerosis. The prevalence in women aged 65 corresponding to men 11–13 years younger and these associations remained after extensive multivariable adjustment.^14^ The relative importance of HCC for CACs was significant and comparable to established cardiovascular risk factors.

The higher CAC scores seen in men are likely influenced by long-term exposure to cardiovascular risk factors such as hypertension, diet, lipids, and smoking.^8.^ Smoking shows a notable connection with CAC in both sexes. However, the smoking pattern has radically changed in Sweden last decades and is nowadays very low or around 5 %, likewise the number of reported smokers in this cohort. But the negative health effect of being a smoker previous lingers on for many years after smoking cessation. For the studied cohort in this study the smoking data probably reflect past habits, meaning those who smoked previously and quit, were nevertheless classified as smokers in the analysis of the study results. Increased concentrations of LDL cholesterol and triglycerides, and their correlation with higher CAC scores, were observed in both men and women in the univariate analysis. Waist circumference over 88 cm for women and 102 cm for men, along with hypertension, high cholesterol, and type 2 diabetes. In multivariate regression analysis, after adjusting for all significant CVD risk factors, women with higher CAC scores were strongly associated with age, elevated HCC, smoking, waist circumference over 88 cm, type 2 diabetes, hypertension, and high cholesterol. A similar pattern was observed in men, though high HCC and waist circumference over 102 cm were not significantly linked to higher CACs.

No differences in CACs were observed among men concerning long-term cortisol levels, whereas differences were found among women. Another study showed that urine cortisol levels correlated with a greater increase in CAC among women but not men. Our research confirms these results.^37^ However, urine cortisol does not offer retrospective data on cumulative cortisol levels over months, like for hair cortisol.

Another possible explanation of the sex differences in this resepct is that CACs in men reflect long-term exposures that are not affected by current levels of long-term stress, as indicated by cortisol concentrations. Men experiencing stress, smoking, or poor nutrition are at risk of increased CACs. In contrast, women who face ongoing long-term stress might be more susceptible to stress during midlife and thus show higher CAC levels due to greater stress exposure. Additionally, genetic factors may contribute, as some individuals exhibit a more intense and prolonged cortisol response, leading to a heightened stress reaction. Their bodies tend to overreact, and cortisol levels take longer to return to normal and in this concept it could be of importance for the impact and effect a higher HCC might have on the blood vessels and CAC.^57,58^

Age influences arterial CAC in both genders. In this study, participants aged 55-65 showed a strong association with high CAC scores in women and men alike. Among the oldest group (60-65 years), the odds ratio (OR) was 2.63 for men and 2.45 for women. Women with lower educational levels had a 26% increased risk of high CACs, though this was not statistically significant. Perceived stress and irregular exercise did not significantly affect CAC levels in either sex, although perceived stress in some respects correlated with a higher risk for women, possibly because most participants fell into this category. Other research indicates that low physical activity, high-fat diets, and smoking contribute to CVD.^8^ Smoking was a clear risk factor for CAC in both sexes and was directly associated with high scores. However, the smoking pattern has radically changed in Sweden last decades and is nowadays very low, but the negative health effect of being a previous smoker lingers on.The study did not include nutritional data that might influence CAC, likely due to the difficulty in obtaining long-term dietary information in the context of artery calcification, which develops over years and is common among older adults. All participants diagnosed with hypertension and high cholesterol were under medication, but yet they showed elevated cortisol levels.

### Relevance

Our research indicates that primary care should view long-term stress as a potential risk factor for cardiovascular disease in middle-aged women. Healthcare professionals must pay close attention to cardiovascular risks linked to stressors and take nonspecific symptoms like chest pain seriously. Preventive approaches might include lifestyle changes, stress management, and risk factor control tailored for this group. Elevated stress levels can trigger cortisol release, which may elevate cardiovascular risk through mechanisms such as atherosclerosis or impacts on heart cells. Although coronary artery calcification naturally progresses with age, it generally occurs about 10-15 years later in women compared to men. Recognizing long-term stress as a potential cardiovascular risk factor is becoming increasingly crucial.

### Strengths and Limitations

The key strengths of this study are its large sample size and its population-based approach. Few similar studies of this scale exist, one exeption is the Whitehall II study in the U.K.^30^ However, the SCAPIS pilot study indicated potential selection bias, with lower participation rates among citizens with low socioeconomic status.^46^

Our study’s unique contribution lies in including CAC measured through a multi-slice computed tomography scanner, which is a notable strength. Other research indicates that stress responsivity correlates with higher CAC. Therefore, cortisol might play a role in both structural and functional heart adaptations, as well as in the development of atherosclerosis. Consequently, methods that assess the effects of cumulative psychosocial exposure could be crucial for evaluating CAC risk. HCC reflects average cortisol over weeks or months, atherosclerotic processes indicated by CAC develop slowly, often spanning longer than the period represented by HCC at sampling. Therefore, presuming chronically elevated cortisol levels existed before the three months prior to sampling cannot be confirmed. Since this study is a cross-sectional study there is a temporal disconnect between time for measuring hair cortisol levels and CACs and this fact is a limitation in the study. It´s not clear if the studied 3 months of HCC data is sufficient to capture the process of atherosclerosis development. Cortisol in hair is measured as mean levels during a retrospective 3-month period, while CACs is measured at a certain time point, but the calcification process principally increase over time.

Methodologically, in this study, CACS was divided into three categories, in accordance with a previous SCAPIS study, which reported that the CACS distribution was skewed. Therefore, this variable was categorized into CACS ≥100 and CACS 1–99, with both categories compared with CACS = 0. This categorization may be considered a strength, as it captures all individuals with a CACS above 0, despite the small number of participants in the highest score category in this sub study, which included a total of only 4,921 participants. It would have been preferable to use more detailed categories if hair samples had been available from all 30,000 participants in the larger SCAPIS study. However, although the same study protocol was used at all study sites, optional examinations could be added depending on local research interests; hair samples were collected at only two sites. Therefore, we can only speculate about what the results might have been if both HCC and CACS had been measured in all participants.

A methodological challenge in measuring hair cortisol is that steroid medication can cause elevated cortisol levels. The cohort studied included 100 participants who regularly used steroid ointments and creams. This group showed significantly higher UTHCC values, but these higher levels might also be attributed to their medication use. To reduce this potential bias, this small subgroup of 100 participants was excluded from further analysis.

A common limitation in studies measuring hair cortisol is that mostly men with sufficient hair length can be included, excluding those with insufficient or short hair. This could potentially impact the results if hair length or absence is linked to long-term stress, though no evidence currently suggests this as a confounder. An earlier analysis of non-attendees, i.e. men without hair samples compared to those with samples, in the SCAPIS cohort showed no significant differences in educational level or cardiovascular risks.^44^ The RIA method’s limitation is that it can produce very high cortisol values in about 2–3% of the population, but the cause of these elevated readings remains unclear.^55^ This allows comparisons between groups rather than relying solely on traditional diagnostics to set reference intervals. All women in this study are over 50, having entered menopause, which may influence cortisol levels and cardiovascular risk.^46^ The study accounts for menopause and hormone use, such as estrogen, which could impact HCC levels, this is considered as a strength (Tables 2 and 3).

Since this is a cross-sectional study, we cannot draw causal conclusions about HCC and CACs, only that an association is evident for middle-aged women. Additionally, although HCC reflects average cortisol over weeks or months, the atherosclerotic processes indicated by CACs develop slowly, often spanning longer than the period represented by HCC at sampling. Therefore, it’s difficult to establish any temporal relationship between hair cortisol levels and atherosclerosis. Chronically elevated cortisol levels might exist before the three months prior to sampling but cannot be confirmed. A minor limitation in this study is that we could not determine how various hair treatments might influence HCC. Nonetheless, natural hair color did not show any differences in HCC (**see** Table S1). Additionally, factors such as hair color and washing frequency had only minimal effects on our radioimmunoassay method used to measure HCC.^56,59^

## Conclusions

We found a positive association between HCC and CAC score among middle-aged women, but not in men. This indicates that prolonged stress exposure impacts cardiovascular risks more significantly in middle-aged women than in men. The relative importance of HCC for CACs was significant and comparable to established cardiovascular risk factors. However, additional research is necessary to determine any causal relationship and the underlying reasons for these sex differences.

## Supplemental Material

Supplemental Material - Increased Hair Cortisol Levels Is Associated With Coronary Artery Calcification in Middle-Aged Women but Not in Men Supplemental Material for Increased Hair Cortisol Levels Is Associated With Coronary Artery Calcification in Middle-Aged Women but Not in Men by Fredrik Iredahl, Elvar Theodorsson, Tomas Faresjö, Michael P. Jones, Andrea Lebena, Per Dahlqvist, Åshild Faresjö in Chronic Stress

## Data Availability

The data that support the findings of this study are available on request from the corresponding author: ashild.olsen.faresjo@liu.se. The data are not publicly available due to privacy or ethical restrictions and are stored at the repository at Linköping University Electronic Press https://ep.liu.se.

## Appendix

### Abbrevations

CACs: Artery calcification score
BMI: Body mass index
CVD: Cardiovascular disease
RIA: Competitive radioimmunoassay
CHD: Coronary heart disease
HCC: Hair cortisol concentrations
HDL: High-density lipoprotein
HPA: Hypothalamic-pituitary-adrenal axisIQRinterquartile range
LogHCC: Logarithmically transformed hair cortisol concentrations
LDL: Low-density lipoprotein
MI: Myocardial infarction
SoHT: Society of Hair Testing
SCAPIS: Swedish Cardio Pulmonary bioImage Study
UTHCC: Untransformed hair cortisol concentrations
