# Supplementary material for: Increased Hair Cortisol Levels Is Associated With Coronary Artery Calcification in Middle-Aged Women but Not in Men
Source: Chronic Stress (Thousand Oaks). 2026 Aug 11;10:24705470261476161. doi: 10.1177/24705470261476161 (PMC13462509; doi:10.1177/24705470261476161)
Supplement: Supplementary file 1 [file sj-pdf-1-css-10.1177_24705470261476161.pdf]

Supplement Data (for publications)

Table S1. Analysis of women participants in the highest untransformed Hair cortisol concentration compared to those in the low/medium.*

**Variables**	**Low/medium > 75 pg/mg HCC n=2522**	**High < 75 pg/mg HCC n=615**	**p-value**
Age-group	% (n)	% (n)	0.5
50-54 years	35.5 (895)	33.3 (205)	
55-59 years	32.2 (811)	32.5 (200)	
60-64 years	32.4 (816)	34.1 (210)	
Education			0.9
Low	7.7 (190)	7.4 (44)	
Medium	45.7 (1128)	45.2 (270)	
High	46.6 (1150)	47.4 (283)	
Civil status			0.3
Married/co-habited	78.5 (1935)	76.7 (458)	
Singel/unmarried	21.5 (530)	23.3 (139)	
Hair colour			0.7
Blond /grey	73.7 (1589)	74.4 (422)	
Black /brown /red	26.3 (560)	25.6 (145)	
Physical activity			0.6
No exercise or little	40.2 (972)	41.4 (242)	
Exercise (regular)	59.8 (1444)	58.6 (342)	
Perceived stress			0.8
Always Stressed	22.8 (558)	23.2 (136)	
Never stressed	3.4 (82)	3.9 (23)	
Smoking			0.6
Yes, now and previous	44.0 (1071)	45.1 (264)	
Waist circumference			0.2
>88 cm	46.2 (1165)	50.0 (307)	
80-88 cm	26.8 (677)	24.9 (153)	
BMI			0.03
Overweight	36.9 (916)	36.7 (222)	
Obese	18.9 (469)	24.6 (149)	
Low HDL outside reference range	6.5 (163)	6.0 (37)	0.6
High LDL outside reference range	8.6 (216)	10.5 (67)	0.15
High triglycerides outside the reference range	3.7 (94)	4.7 (29)	0.2
Hypertension, yes	19.6 (481)	26.1 (155)	<0.001
High cholesterol	7.8 (192)	10.4 (62)	0.04
Diabetes type 2	4.5 (114)	7.2 (44)	0.02
Impaired glucose balance	13.9 (350)	12.4 (76)	
Use of estrogen during menopause	19.9 (483)	21.0 (124)	0.5

***Based on thousands of hair cortisol analyses made in the laboratory among adults.**

S2. Model 1, 2 and 3. The regression model 1 comprised sociodemographic data, model 2 comprises lifestyle data and finally model 3 comprise medical condition. Separate analyses were undertaken for women and men. Possible factors for higher CAC score among SCAPIS *Female* study population. Dependent variable CACs score=0, CACs score=1 (1-99), and CACs score >100.

Factors	Wald	p-value	OR	95% confidence interval Lower bound	Upper bound
**Model 1:**					
**Log-cortisol**	5.33	0.02*	1.09	1.01	1.17
**Education low vs high**	17.12	<0.001*	1.84	1.03	2.45
**Education medium vs high**	15.10	<0,001*	1.40	1.19	1.67
**Age 60-69 years vs <55 years**	116.93	<0.001*	3.13	2.53	3.82
**Age 55-59 years vs <55 years**	43.61	<0.001*	2.03	1.65	2.53
**Civil status: Single vs partnered**	3.14	0.76	0.84	0.70	1.02
**Model 2:**					
**Log-cortisol**	7.69	0.006	1.11	1.03	1.19
**Perceived stress: Always vs never**	0.12	0.72	0.91	0.56	1.50
**Perceived Stress now and then vs never**	0.52	0.47	1.19	0.74	1.90
**Smoking: Yes, vs No**	55.62	<0.001	1.89	1.60	2.23
**Exercise: No vs Yes**	6.19	0.013	1.25	1.05	1.49
**Waist circumference**					
**>88 cm vs <80cm**	18.33	<0.001*	1.29	1.29	1.97
**80-88 cm vs <80cm**	1.76	0.19	1.17	0.92	1.49
**Use of oestrogen yes vs no**	0.000	0.99	1.00	0.81	1.23
**Model 3:**					
**Log-cortisol**	4.18	0.04*	1.08	1.00	1.16
**Diagnosed Diabetes***	9.74	0.002*	1.75	1.23	2.50
**Diagnosed impaired glucose***	8.21	0.004*	1.39	1.11	1.74
**Diagnosed Hypertension***	31.59	<0.001*	1.74	1.43	2.11
**Diagnosed High cholesterol***	22.63	<0.001*	1.96	1.49	2.60
**HDL** outside reference range (low)	2.31	0.13	0.76	0.53	1.08
**LDL** outside reference range (high)	1.04	0.31	0.86	0.64	1.15
**Triglycerides** outside reference range (high)	0.36	0.55	1.14	0.74	1.74

Model 1. intercept: -2 log likelihood: 4212,90, df:6, p<0.001. Cox and Snell: 0.055 and Nagelkerke: 0.071. **Model 2:** intercept: -2 log likelihood: 3992,50, df:8, p<0.001. Cox and Snell: 0.039 and Nagelkerke: 0.051. **Model 3:** intercept: -2 log likelihood: 4118.40, df:8, p<0.001. Cox and Snell: 0.037 and Nagelkerke: 0.048.

*vs not diagnosed with indicated condition

S3. Model 1, 2 and 3. The regression model 1 comprised sociodemographic data, model 2 comprises lifestyle data and finally model 3 comprise medical condition. Separate analyses were undertaken for women and men. Possible factors for higher CAC score among SCAPIS *male* study population. Dependent variable CACs score=0, CACs score=1 (1-99), and CACs score >100.

Factors	Wald	p-value	OR	95% confidence interval Lower bound	Upper bound
**Model 1:**					
**Log-cortisol**	0.027	0.87	1.01	0.92	1.10
**Education low vs high**	0.009	0.92	0.98	0.68	1.41
**Education medium vs high**	0.42	0.52	1.07	0.87	1.31
**Age 60-69 years vs <55 years**	70.51	<0.001*	2.81	2.21	3.58
**Age 55-59 years vs <55 years**	12.91	<0.001*	1.55	1.22	1.97
**Civil status: Single vs partnered**	0.32	0.57	1.07	0.84	1.36
**Model 2:**					
**Log-cortisol**	0.13	0.72	1.02	0.93	1.11
**Perceived stress: Always vs never**	1.43	0.23	0.76	0.49	1.19
**Perceived Stress now and then vs never**	1.48	0.22	0.79	0.53	1.16
**Smoking: Yes, vs No**	16.98	<0.001*	1.53	1.25	1.86
**Exercise: No vs Yes**	0.045	0.83	1.02	0.83	1.25
**Waist circumference**					
**>102 cm vs <94cm**	17.57	<0,001*	1.98	1.44	2.73
**94-102 cm vs <94cm**	3.91	0.048*	1.36	1.00	1.83
**Model 3:**					
**Log-cortisol**	0.009	0.92	1.00	0.90	1.09
**Diagnosed Diabetes***	20.92	<0.001*	2.32	1.62	3.34
**Diagnosed impaired glucose***	8.31	0.004*	1.50	1.14	1.98
**Diagnosed Hypertension***	46.26	<0.001*	2.35	1.84	3.01
**Diagnosed High cholesterol***	19.09	<0.001*	2.00	1.47	2.73
**HDL** outside reference range (low)	0,007	0.93	1.02	0.68	1.52
**LDL** outside reference range (high)	3.18	0.07	0.73	0.51	1.03
**Triglycerides** outside reference range (high)	1.11	0.26	0.61	0.74	1.67

Model 1. intercept: -2 log likelihood: 3042.76, df:6, p<0.001. Cox and Snell: 0.054 and Nagelkerke: 0.073. **Model 2:** intercept: -2 log likelihood: 3007.84, df:7, p<0.001. Cox and Snell: 0.031 and Nagelkerke: 0.036. **Model 3:** intercept: -2 log likelihood: 2959,85 df:8, p<0.001. Cox and Snell: 0.091 and Nagelkerke: 0.104.

*vs not diagnosed with indicated condition

SCAPIS (Swedish Cardio Pulmonary bioImage Study) some general information

As of July 2026, more than 300 articles based on SCAPIS data have been published.

Data for this sub-study were obtained from the Swedish CArdioPulmonary bioImage Study (SCAPIS), the largest medical research initiative conducted in Sweden, comprising 30,154 participants recruited from six university hospital sites. Participants were randomly selected from the Swedish general population and were aged 50–64 years. A standardized study protocol was applied at all sites, although optional examinations were permitted according to local research interests. Hair cortisol measurement was included as part of a local research initiative. Hair samples were collected exclusively at two designated sites and only from individuals within the specified age range.

Recruitment took place between 2014 and 2018. During this period, participants underwent comprehensive assessments over three days, including questionnaires, clinical and physiological examinations, and blood sampling. At each site, 5,000–6,000 inhabitants were contacted, and recruitment continued until the target sample size (N = 30,154) was reached. SCAPIS was specifically designed to improve understanding of cardiovascular and pulmonary diseases through detailed imaging and extensive biomarker profiling.

An analysis of individuals in the SCAPIS cohort who did not participate (i.e., answered “no” to participation) showed no significant differences in educational background or cardiovascular risk factors compared with those who participated. However, differences were observed with respect to socioeconomic factors. Data on non-response in this sub-study are limited to the two locations where hair samples were collected.

Some publications:

Yvonne Natt och Dag, Gunnar Engström2 and Maria Rosvall. Cross-sectional associations of optimism with artery calcification and function: The SCAPIS study. Journal of Public health Research. 2022; Vol 11 (2)1-8.doi: 10.1177/22799036221110021.

Björk J, Strömberg U, Rosengren A, Toren K, Fagerberg B, Grimby-Ekman A. Predicting participation in the population-based Swedish cardiopulmonary bio-image study (SCAPIS) using register data. Scand J Public Health. 2017;45(17 suppl):45–9. doi: 10.1177/1403494817702326

Natt o Dag Y, Engström G, Rosvall M. Life satisfaction and coronary atherosclerosis: The SCAPIS study. J. Psychosom. Res. 152 (2022) 110663. doi: 10.1016/j.jpsychores.2021.110663

Bergström G, Persson M, Adiels M. et al. Prevalence of Subclinical Coronary Artery Atherosclerosis in the General Population. Circulation. 2021;144(12):916-929. doi: 10.1161/circulationaha.121.05534

Söderberg M, Eriksson H, Torèn K, Bergström G, Andersson E, Rosengren A. Psychosocial job conditions and biomarkers of cardiovascular disease: A cross-sectional study in the Swedish CArdioPulmonary bioImage Study (SCAPIS). Scand J Public Health. 2023 Aug;51(6):843-852. doi: 10.1177/14034948211064097

Östgren CJ, Söderberg S, Festin K. et al. Systematic Coronary Risk Evaluation estimated risk and prevalent subclinical atherosclerosis in coronary and carotid arteries: A population-based cohort analysis from the Swedish Cardiopulmonary Bioimage Study. Eur J Prev Cardiol. 2021;28(3):250-259. doi: 10.1177/2047487320909300

Swahn E, Lawesson SS, Alfredsson J. et.al. Sex differences in prevalence and characteristics of imaging-detected atherosclerosis: a population-based study. Eur Heart J Cardiovasc Imaging 2024; 25: 1663–1672. doi: 10.1093/ehjci/jeae217

Faresjö Å, Theodorsson E, Stomby A. et.al. Higher hair cortisol levels associated with previous cardiovascular events and cardiovascular risks in a large cross-sectional population study. BMC Cardiovasc Disord. 2024;24(1):536. doi: 10.1186/s12872-024-04221-2

Wuopio J, Ting LY, Dekkers KF et.al. The metabolic signature of salt intake: a cross-sectional analysis from the SCAPIS-study. Nutrition & Metabolism (2025) 22:104 https://doi.org/10.1186/s12986-025-00997-y
